# Indents

**Published:** 1912-02-15

**Authors:** 


### INDENTS.

**☞Brief Articles of Technical or Scientific Value will receive notice and comment. Contributions invited.**

*Cocain or Substitutes in Dental Operations*

Every operator who is familiar with local anesthesia knows that in the selection of anesthetics he should endeavor to avoid or overcome the following untoward complications: First, insufficient anesthesia; second, post-operative pain of continued duration, which renders the value of local anesthesia rather problematic; third, persistent secondary hemorrhage, which frightens the patient and is inconvenient for the operator; fourth, idiosyncrasies and toxic effects. The author formerly employed very small doses of 0.1 cocain solution in 100 gm. of physiologic salt solution, with or without the addition of from 1 to 20 drops of a 1:1000 suprarenal extract solution, for surgical operations. In dental operations, however, he found that in most cases a 0.5 per cent. solution is required, which produces toxic symptoms in the heart and the nervous system, most dental patients being in a run-down condition owing to their unwisely prolonged waiting and their intense fear of dental operations. The dentist is at a disadvantage as compared with the surgeon, as he is invariably blamed for any failure or accident in local anesthesia, and any intoxication or sexual irritation following the use of local anesthetics unjustly brings him into conflict with the medico-legal code. For the latter reasons, many dentists employ general anesthesia much more frequently than is necessary or even justified. Of the substitutes for cocain, namely novocain and alypin the former has proved to be considerably less toxic, and the author has not observed one single case of untoward results or necessity for antidotes in eighteen months' frequent application of these substitutes, while after cocain injection toxic symptoms were frequently noted. Discrimination in the use of local anesthesia is necessary; in very frail and timid patients general anesthesia is preferable, while in patients with weak or diseased hearts local anesthesia is contraindicated. In large surgical clinics, which are now using novocain exclusively, untoward after-effects are very rarely noted. In children only substitutes of cocain should be employed. Post-operative pain, which after cocain injection was the rule, is entirely absent after the application of novocain, while in regard to post-operative hemorrhage the results are about equal. While there is no doubt that cocain is the strongest anesthetic, its substitutes, especially for injection anesthesia, are preferable, and their general adoption should be heartily encouraged. For pressure anesthesia and as an admixture to pulp devitalizing agents, cocain still holds its own.—Dr. Duschinsky, *Cosmos*.

*A Spartan System of Hygiene.* Radically differing from the plan of voluntary enrollment of patients by the physician of their choice, is that presented by Benjamin Moore, of Liverpool. He contemplates the organization of a Bureau of Health, the whole work of preventing disease and caring for the sick to be placed in the hands of the government, and the expenses defrayed by general taxation. Medical treatment would thereupon be free and compulsory. He is quite sanguine to believe that ten years of this system would rid the country of nearly all need for practicians as healers; medical officers being left free to devote themselves to educating the public in sanitation and its enforcement, with the abolition of infectious diseases. There would be work enough for the 32,000 medical men on the British register, whose labors at present are almost wasted because they are misdirected, owing to the present methods of attacking disease, which are medieval in their antiquity and ignorance. His plan comprises a system of state insurance against sickness, and he computes that the present cost would amply provide for the entire expense.

We trust the day is remote when such a plan could be adopted in free America; when the secrets of the sick-room now entrusted to the medical advisor in whom one places confidence should be made a matter of public record by a government official. We doubt whether there is a civilized land so official-ridden as to render this possible. Even in well-harnessed Germany the overwhelming flood of quacks shows the impulse of the individual to assert his right of choice of a medical adviser.

Curiously, *The Pasadena Times* takes our own editorial, advising level yearly payments to the physician of choice, he to assume the role of sanitary director and prevent disease as well as treat it; and against this *The Times* directs an outburst of indignation that would strike the bullseye were aimed against Moore's plan. We suspect our esteemed lay contemporary got his files mixed, and affixed his diatribe against Moore to our own editorial. Certainly nothing in the latter so much as intimated govermental control or limitation of the individual in his choice of a physician.—*Am. Journal Clinical Medicine.*

*Technical versus the Scientific.* If it be true that dentistry in general must halt a certain objectionable practice —viz, that when some method, logical deduction, treatise, or book is brought fourth—perchance before an assembly such as this—several men stigmatize it as not being practical. There is nothing practical in my opinion that is not scientific; nothing scientific that is not true. If we hold ourselves up as a profession we must get rid of this mechanical estimate—this antagonism to the scientific. These are days of education, knowledge, and advancement, and we must drop the idea that a dentist must have brains only in his fingers. It is about time that the college teachers and professors correct that erroneous idea. We must be organized into an independent dental society, having control not alone of its own journal but of every thing that pertains to the true uplift and advancement of the profession—Dr. Flanagan, in *Cosmos.*

